# How Many Tree Species of Birch Are in Alaska? Implications for Wetland Designations

**DOI:** 10.3389/fpls.2020.00750

**Published:** 2020-06-11

**Authors:** Carol A. Rowe, Robert W. Lichvar, Paul G. Wolf

**Affiliations:** ^1^Department of Biology, Utah State University, Logan, UT, United States; ^2^United States Army Corps of Engineers, Cold Region Research and Engineering Laboratory, Hanover, NH, United States; ^3^Department of Biological Sciences, The University of Alabama in Huntsville, Huntsville, AL, United States

**Keywords:** *Betula*, *Betula neoalaskana*, *Betula kenaica*, *Betula papyrifera*, ddRADseq, population genomics

## Abstract

Wetland areas are critical habitats, especially in northern regions of North America. Wetland classifications are based on several factors, including the presence of certain plant species and assemblages of species, of which trees play a significant role. Here we examined wetland species of birch (*Betula*) in North America, with a focus on Alaska, and the use of birche tree species in wetland delineation. We sampled over 200 trees from sites, including Alaska, Alberta, Minnesota, and New Hampshire. We used genetic data from over 3000 loci detected by restriction site associated DNA analysis. We used an indirect estimate of ploidy based on allelic ratios and we also examined population genetic structure. We find that inferred ploidy is strongly associated with genetic groupings. We find two main distinct groups; one found throughout most of Alaska, extending into Alberta. This group is probably attributable to *Betula kenaica, Betula neoalaskana*, or both. This group has a diploid genetic pattern although this could easily be a function of allopolyploidy. The second major genetic group appears to extend from Eastern North America into parts of southeastern Alaska. This group represents *Betula papyrifera*, and is not diploid based on allelic ratios. Published chromosome counts indicate pentaploidy. Because *B. papyrifera* is the only one of the above species that is distinctly associated with wetland habitats, our findings indicate that tree species of birch found in most parts of Alaska are not reliable indicators of wetland habitats. These results help to support stronger wetland ratings assigned to the tree species of birch for delineation purposes.

## Introduction

Wetland areas are some of the most biologically productive habitats, and they provide several vital services for the environment and for humans (Finlayson et al., 2005; Junk et al., 2013). In the United States, wetland delineation is based on three factors: vegetation, soils, and hydrology, following protocols described in the United States Corps of Engineers Wetland Delineation Manual (Environmental Laboratory, 1987), in addition to those of appropriate regional supplements. To determine whether vegetation is predominantly hydrophytic or not, plant species are assessed using wetland indicator status ratings on the National Wetland Plant List (NWPL) (Reed, 1988; Lichvar et al., 2016). The combination of wetland ratings and percent cover are used in a formula to determine if an area meets the requirements for the presence of hydrophytic vegetation (Lichvar et al., 2012). Thus, wetland delineation is dependent on correct identification of species presence, which, in turn, depends on reliable taxonomy. Here we examine species relationships among arborescent taxa of *Betula* in Alaska, some of which are wetland indicator species.

The state of Alaska includes over 700,000 km^2^ of wetlands (Hall et al., 1994), which accounts for approximately 8% of global wetland areas (Whitcomb et al., 2009). Much of Alaska’s wetlands are dominated by trees, especially birch (*Betula*) species. Because of the potential contribution of percent cover in determining hydrophytic vegetation for delineation purposes, birch trees often affect the outcome of wetland delineation determination. The most recent treatments of Alaskan birches denote three tree species (Hultén, 1968; Furlow, 1997; Packee, 2004): *Betula papyrifera, Betula kenaica*, and *Betula neoalaskana.* Although Furlow (1997), in Flora of North America (FNA), discusses *B. papyrifera* in Alaska, the distribution map only shows the species in the southeastern part of the state. Wetland ratings for species range nationally from obligate wetland to upland. In Alaska before 2016, *B. papyrifera* had a facultative (FAC) wetland rating, whereas the other two species are considered facultative upland (FACU) species (Lichvar et al., 2016). Thus, being able to identify birch trees to species has implications for wetland delineation in Alaska. However, morphological distinctions among these tree species are subtle, and the limited distribution of *B. papyrifera* in Alaska has often been overlooked, which may have led to further confusion. *B. kenaica* is distributed across central Alaska and south to Kenai Peninsula, and barely into Yukon, whereas *B. neoalaskana* is found throughout much of Alaska, except the northern and western regions, as well as across much of Yukon, Northwest Territories, Alberta, Saskatchewan, and Manitoba. Table 1 outlines the key morphological features of Alaskan tree birches based on descriptions by Viereck and Little (2007) and summarized by Packee (2004). A genetic approach would help to verify the distribution and taxonomy of birches in and beyond Alaska, especially in the light of morphological confusion. Note that several upland dwarf birches (e.g., *Betula nana*) are also found in Alaska, and some of these are used in wetland rating systems, but these species are not the focus of the current study.

**TABLE 1:** Morphological characters used to distinguish the three species of *Betula* found in Alaska, based on descriptions by Viereck and Little (2007) and summarized by Packee (2004).

**Character**	***B. kenaica***	***B. papyrifera***	***B. neoalaskana***
Mature bark	Thinly layered; red/brown to gray/white with pink tinges. Lenticels black and prominent	Cream to chalky white, thick layers. Lenticels pale	Red to off-white, thick layers. Lenticels black and prominent
Young bark	Dark; glabrous	Reddish, coppery, or purple, dark to light; glabrous	Dark rough with resin glands
Spur shoots	6–15 mm	6–15 mm	6–15 mm
Buds	Greenish or dark brown; <10 mm	Greenish or dark brown; <10 mm	Greenish or dark brown; <10 mm
Leaves	Triangular; 4–5 cm long, 2.5–4.5 cm wide	Ovate; 5–9 (12) cm long, 4–7 cm wide	Oval to somewhat triangular; 4–8 cm long, 2.5–5 cm wide
Leaf margin	Doubly serrate, often with fringe of light hairs	Doubly serrate or serrate-dentate	Coarsely doubly serrate, no hairs
Leaf base	Rounded to almost flat	Rounded, cuneate, or truncate	Broadly cuneate to round
Infructescence	2–5 cm	2.5–5 cm	2–4 cm

Chromosome numbers in *Betula* are variable, but appear to build on a base haploid number of 14 (Woodworth, 1929; Taper and Grant, 1973). Following the FNA treatment (Furlow, 1997), *B. papyrifera* is known with 2*n* = 56, 70, and 84; *B. neoalaskana* has 2*n* = 28; and *B. kenaica* with 2*n* = 70. However, given the variation within species with multiple counts, and the wide geographic ranges involved, it is likely that additional cytogenetic variation exists.

Several previous systematic and population studies have focused on *Betula*. The genus is found throughout temperate regions of the northern hemisphere, extending north of the Arctic Circle. *Betula* is treated as about 30–120 species (De Jong, 1993; Furlow, 1997; Ashburner and McAllister, 2013; Wang et al., 2016). At the genus level, relationships among species and subgenera have been examined using rRNA genes (Li et al., 2005) and nucleotide variation in nitrate reductase (Li et al., 2007). Previous studies have also examined relationships using variation at nuclear *ADH* and plastid *matK* loci (Järvinen et al., 2004) and also amplified fragment length polymorphisms (AFLPs, Schenk et al., 2008). All these studies found distinct patterns at the generic level, with clear separation of most subgenera. However, at the species level, hybridization and introgression appears to be a common thread (Wang et al., 2013, 2016; Zohren et al., 2016; Tsuda et al., 2017). One study, using microsatellite data (Thomson et al., 2015), focused on North American *Betula*, finding distinct geographic groupings and a signature of recent introgression. However, as far as we are aware, wide sampling from Alaska has not been included in any genetic based study of *Betula*.

Using *Betula* as a wetland indicator requires better knowledge of the relationships among, and distributions of, Alaskan taxa, as well as affinities to *Betula* outside Alaska. Here we used genetic tools to examine groupings of *B. papyrifera, B. neoalaskana*, and *Betula kenaika.* We used double digest Restriction-Site Associated DNA sequencing (ddRADseq) of Alaskan tree birches to determine: (1) if there are distinct genetic groups of *Betula* within Alaska; (2) whether any genetic groupings match taxonomy, and (3) to examine genetic relationships among *Betula* samples in Alaska and other parts of North America.

## Materials and Methods

### Sampling

**FIGURE 1:**
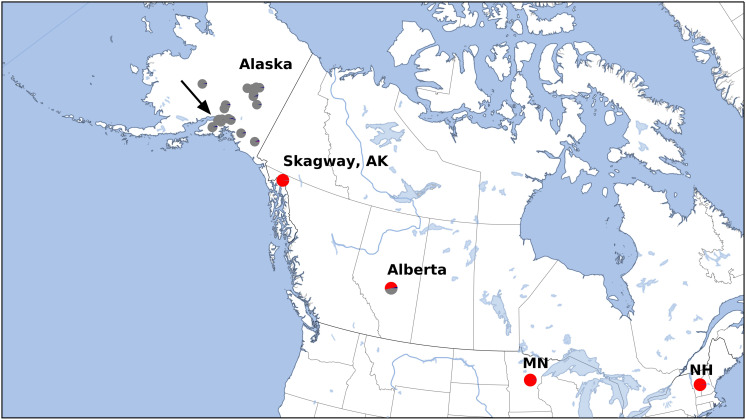
Sample locations for this study. See Supplementary Table 1 for detailed locations. Arrow indicates higher scaled map of region around Anchorage, Alaska (Figure 2). Colors correspond to Structure results (Figure 3), which also corresponds with estimated cytogenetic grouping.

We sampled 5–10 trees from each collection site, which in general was isolated from others by at least 100 km, and trees within a site were at least 1 km apart. In a few areas of Alaska, we increased the sampling density to explore what appeared to be higher levels of morphological variation. We sampled at lower density outside Alaska to determine relationships across *B. papyrifera* in North America. In total, we sampled 202 plants, from 36 sites (Supplementary Table 1 and Figure 1). Samples collected in Alberta are vouchered at ALTA (Vascular Plant Herbarium, University of Alberta). The remainder are at UTC (Intermountain Herbarium at Utah State University). We examined our voucher specimens for morphological variation, including the characters listed by Furlow (1997): leaf shape, base, and margin, bark color and texture, and infructescence shape.

### Genomic Library Preparation

We extracted DNA using the Qiagen DNeasy 96 Plant Kit (Cat. No. 69181; Qiagen Inc., Valencia, CA, United States). We prepared a genomic library following a ddRADseq protocol (Gompert et al., 2012; Parchman et al., 2012). We used EcoR1 and Mse1 to digest genomic DNA, then ligated the *Eco*RI ends of fragments to barcoded (indexed) oligonucleotides (with barcodes unique to each individual), and a standard, non-barcoded oligonucleotide to the *Mse*I ends. We then PCR-amplified samples using iproof high-fidelity DNA polymerase (New England Biolabs) with primers that overlap the ligated oligonucleotides. The library was then reduced to fragments in the size range of 250–350 bp using a Blue Pippin (Sage Science, Beverly, MA, United States). Quality and quantity were further verified using TapeStation 2200 (Agilent Technologies). The size-selected, multiplexed samples were run on a single lane of Illumina HiSeq 2500 with 100 bp single-end reads.

### DNA Data Processing

We processed the raw Illumina reads with ipyrad v.0.7.28 (Eaton, 2014). This program is a series of tools for assembling ddRADseq reads and extracting genotype data. First, within-sample clusters are generated using USEARCH (Edgar, 2010), and reads are aligned using MUSCLE (Edgar, 2004). Error rate and heterozygosity are then estimated, and consensus bases are called and filtered. Finally, clusters are generated across samples to call variants, and filters are applied to the resulting genotype data, which is output in several formats. Due to the lack of a reference genome, we assembled the data *de novo* using VSEARCH (Enns et al., 1990) in ipyrad. Variant calling in RADseq data will always face a tradeoff: cluster too high and alleles will be treated as separate loci; cluster too low and separate loci will be treated as alleles. We explored this parameter in a range of 86–95%, with little effect on our downstream analysis. The results are present at clustering threshold of 90% sequence similarity. Output files from ipyrad were then used for further downstream analyses. To test for possible contamination of ddRAD-seq loci, we used BLASTN (Altschul et al., 1990) of the sample with the highest number of SNP loci, against GenBank release 226 (July 9, 2018). We used a match threshold of 1e-05. For the contamination tests we used loci detected in 50% or more of individuals; whereas for downstream population genetic analyses, we used only loci in 70% or more of individuals.

### Analysis of Genetic Structure and Ploidy

To explore genetic structure of samples and regions, we used the program STRUCTURE 2.3.4 (Pritchard et al., 2000; Falush et al., 2003, 2007; Hubisz et al., 2009). We used a burn-in of 100,000 followed by 250,000 iterations with 50 replicates for each value of *K* (2–6) source populations. We determined the optimal number of subdivisions with a statistical measure of goodness of fit using the Bayesian information criterion (BIC) taken across *k* = 1 to *k* = 20. We also used the method of Evanno et al. (2005) to estimate the optimal *k*.

*Betula* species are known to vary for ploidy level (Woodworth, 1929; Taper and Grant, 1973; Brown and Al-Dawoody, 1977). Ploidy can be detected by counting chromosomes or measuring DNA content of cells using flow cytometry. An indirect method is based on the ratio of alleles at heterozygous loci. In a diploid individual, it is expected that each allele is present in equal proportions. Thus, a genomic library is expected to capture similar numbers (read depth) of each allele, whereas triploids, or other unbalanced ploidy levels may have uneven ratios of alleles (e.g., 3:1, 4:1, etc.). This prediction can be tested and used to estimate ploidy variation in ddRADseq data. Here we used the method of Gompert and Mock (2017) to detect samples behaving as genetic diploid versus other possible ploidy levels. We used a burn-in of 500 steps followed by 1000 MCMC iterations, retaining 5 unthinned steps on each iteration. We used a strict criterion to define ploidy: any overlap of 95% equal-tail probability intervals (ETPIs) are classified as ambiguous. We also do not distinguish among different forms of unbalanced polyploidy: we classify samples as diploid (clearly 1:1 ratio), polyploid (ratios other than 1:1), or ambiguous (overlap of ETPIs).

## Results

### Test of Contamination

Our complete data analysis pipeline is available on Github: https://github.com/carol-rowe666/Betula_ddrad. DNA sequence reads are deposited in the NCBI Sequence Read Archive (SRA), accession PRJNA556994. Of the 6453 ddRADseq loci detected in 50% of samples, 1236 had hits to sequences in GenBank, none had a best hit to bacteria, 1230 had a best hit to eukaryota, all of which were green plants (Viridiplantae), and no hits to fungi. Of the Viridiplantae, one was a liverwort (*Nardia*), one was a gymnosperm (*Macrozamia*), and the remainder were angiosperms, of which 558 were Fagales (the order to which *Betula* belongs). These results indicate that our ddRADseq loci are unlikely to contain much contamination, if any. Because the loci are anonymous, we expect to have a large number with no regions of sequence similarity in other organisms. Regardless, the lack of hits to bacteria or fungi combined with the very high number to Fagales suggests very low levels of contamination in our final data set of SNP loci.

### Population Genetic Structure

**FIGURE 2:**
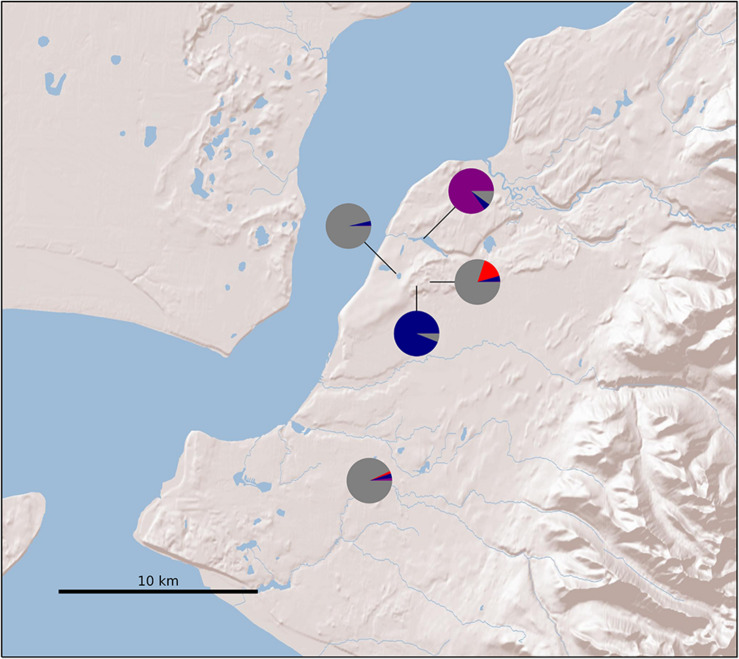
Sample locations in Alaska. Colors correspond to Structure results (Figure 3).

**FIGURE 3:**
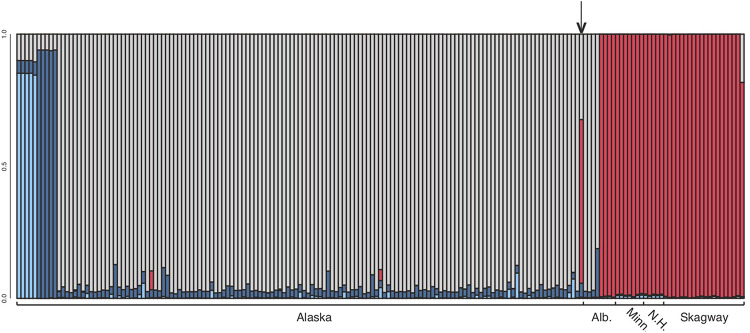
Structure analysis at *k* = 4, which assumes four source populations. Each column represents an individual. Note that the dark and light blue samples (left side) are from 6 and 7 km north of Anchorage, Alaska, respectively (see Figure 2). These samples clustered with remaining Alaska samples at *k* = 2 and *k* = 3. The optimal number of groups was *k* = 2. Note that Skagway (in south-eastern Alaska) groups with other eastern samples (except BP_25 which 81% Eastern, 19% Western), and one Alaskan sample from close to Anchorage (8553b – indicated by arrow) appears to be a hybrid.

After variant calling we removed 21 samples because the read coverage was too low, leaving 181 samples in the analysis. We detected 3375 ddRADseq loci in 70% of these samples. We used these loci to examine population structure. Our samples of *Betula* fall into two main genetic groups with a few notable exceptions (Figures 1, 3): broadly circumscribed Western (gray + blue) and Eastern (red) population groups. The lowest BIC was for two clusters representing Western and Eastern groups. However, the Evanno et al. (2005) method suggested that up to *K* = 4 was slightly more optimal than two populations sources. We therefore explore relationships across these *k* values. When four source populations are assumed (Figure 2), two small additional groups of samples, close to Anchorage, AK, became distinct, but still genetically much closer to other Alaskan samples than to the Eastern group. Reanalyzing with only samples from Alaska did not change these patterns. The rough boundary between the Eastern and Western groups covers parts of southeastern Alaska and Alberta. We calculated Jaccard similarity indices for all pairwise samples. The mean index within the gray and within the red groups is 0.95. Now comparing across groups, the mean index between gray and either blue group (left side of Figure 3) is also 0.95. These values contrast with the mean between the gray and the red group of 0.91. Thus, divergence of the two blue groups from the gray (all within Alaska) is small compared to divergence between red (Eastern) and gray (Western) groups, and is the same as divergence within the gray group. There are a few notable exceptions to the general pattern. Samples from Skagway, in south-eastern Alaska, group with the “Eastern” group, along with those from Minnesota and New Hampshire, with only one plant from Skagway appearing to possess some of the Western genotype (BP_25: 81% Eastern, 19% Western). Samples from Alberta include a mixture of individuals, some with the Eastern genotype, some with the Western genotype. We detected no individual sample in Alberta with a clear mix of the two groups, which would have indicated a hybrid. We did detect one sample (8553b) in Alaska (6 km NE of Anchorage) with a 62% contribution from the Eastern group, suggesting that this tree is a possible hybrid.

### Ploidy Variation in *Betula*

**FIGURE 4:**
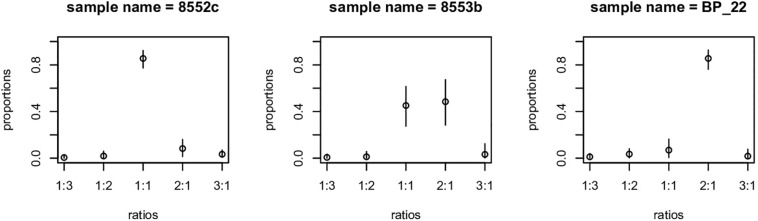
Example of gbs2ploidy output. Sample on left has 1:1 allelic ratio, typical of a diploid or allopolyploidy. Example on the right is indicative of a triploid or higher ploidy level. Example in center is ambiguous – some loci appear to be diploid, whereas others are higher ploidy.

Genetic data can be used to explore ploidy by examining the distribution in allele ratios across large numbers of loci (Gompert and Mock, 2017). Because much higher read depths are required to infer exact ploidy above diploids, we provide only two groupings: “diploids” and what we refer to here as “polyploids.” Note that just because alleles are balanced does not necessarily mean that the plant is diploid, merely that it has diploid genetics; a balanced allotetraploid can have this pattern. What we refer to as polyploidy means that allele depth was skewed, implying an unbalanced ploidy level. Of our 181 samples, 140 appeared to have diploid allele ratios, 26 had polyploid ratios, and 15 samples were ambiguous (Figure 4, Supplementary Table 2, and Table 2). Our analysis detected a complete association between inferred ploidy and genetic groupings, ignoring ambiguous allelic ratios (Figure 2 and Table 2). All samples with the Eastern genotypes (Skagway, some from Alberta, Minnesota, and New Hampshire) had a polyploid ratio of allele depth (26 polyploid and 11 ambiguous), whereas all samples in the Western group had diploid-like ratios (140 diploid and 4 ambiguous). The one sample from Alaska that had an Eastern genetic component was ambiguous for ploidy (Figure 2 and Supplementary Table 2). Some polyploid samples appeared to be 2:1 (indicative of a triploid), some appeared closer to 3:1 (unbalanced tetraploid). These patterns are consistent with observed chromosome numbers indicating that *B. papyrifera* is a pentaploid (Woodworth, 1929; Taper and Grant, 1973).

**TABLE 2:** Structure groupings and ploidy for each population of *Betula.*

**Population**	**State or province**	**Group**	**Ploidy**	***n***
8554	AK	Western	Dip	5
8552	AK	Western	Dip	5
1028	AK	Western	Dip	4
1029	AK	Western	Dip	4
1030	AK	Western	Dip	4
1031	AK	Western	Dip	5
8553	AK	Western*	2 Dip; 2 Amb	4
8555	AK	Western	3 Dip; 1 Amb	4
8556	AK	Western	Dip	4
8557	AK	Western	Dip	3
8558	AK	Western	Dip	5
8559	AK	Western	Dip	5
8560	AK	Western	Dip	5
8561	AK	Western	Dip	5
8562	AK	Western	3 Dip; 1 Amb	4
8563	AK	Western	Dip	5
8564	AK	Western	Dip	4
8565	AK	Western	Dip	5
8566	AK	Western	Dip	3
8567	AK	Western	Dip	3
8568	AK	Western	Dip	4
8569	AK	Western	4 Dip; 1Amb	5
8570	AK	Western	Dip	5
8571	AK	Western	Dip	5
8572	AK	Western	Dip	5
8573	AK	Western	Dip	4
8574	AK	Western	Dip	5
8575	AK	Western	Dip	5
8576	AK	Western	Dip	5
8577	AK	Western	Dip	5
8578	AK	Western	Dip	5
Ken	AK	Western	Dip	2
ALB	Alb	Mixed	4 Dip; 2 Amb; 2 Poly	8
MNSE	MN	Eastern	2 Amb; 2 Poly	7
NB	NH	Eastern	3 Poly; 2 Amb	5
BP	AK	Eastern	16 Poly; 4 Amb	20

*Note one sample from 8553 (asterisk) in Alaska had a significant component of the Eastern group.*

### Morphology

We examined all 71 voucher specimens and focused especially on the characters outlined by Furlow (1997): leaf shape, base, and margin, bark color and texture, and infructescence, if available (Table 1). We were unable to detect any consistent pattern for these or any other characters on the specimens. We focused especially on the eight samples from Alberta, which appeared to represent each of the two distinct genetic groups, but were unable to detect any differences. We also did not observe specimens from Alaska that fell clearly into one of the two species: *B. neoalaskana* and *B. kenaica*.

## Discussion

We detected two distinct genetic, and corresponding cytogenetic, clusters of *Betula*, one (Western) found primarily in Alaska, but extending into Alberta, and the other (Eastern) detected in south-east Alaska, and extending to eastern North America. We infer that the latter refers to *B. papyrifera* and the former refers to *B. neoalaskana* or *B. kenaika*, or both.

### Genetics and Ploidy

The genetic subdivisions detected here were unexpectedly distinct, and the association with inferred ploidy was strong. Based on allelic ratios in our RADseq data, these two groups appear to differ cytogenetically, with *B. neoalaskana*/*B. kenaika* having a diploid genetic pattern, possibly a function of being a balanced allotetraploid, whereas the patterns in *B. papyrifera* are consistent with chromosome counts indicating that it is a pentaploid (Furlow, 1997). The general difference of samples from Alaska and Alberta (Western group) from the Eastern samples is consistent with the patterns detected by Thomson et al. (2015), who observed distinct chloroplast haplotypes in western Canada, in addition to some samples that had haplotypes more common in eastern North America. We find two aspects of these results especially intriguing. One is that we could detect no distinct morphological differences between the two genetic clusters. The other is that we only detected two samples that appeared to combine genotypes between the clusters. The first point is consistent with the complex and confusing taxonomy of *Betula* in Alaska, some treatments of which include *B. papyrifera* throughout the state, not just in the south-east portion. The second point is that hybrids seem to be relatively rare, which is surprising given the morphological similarities. We hypothesize that the two clades are mostly isolated reproductively and that this isolation is maintained by cytogenetic incompatibilities. We only detected two plants with a possible footprint of hybridization: one from near Anchorage and one from Skagway. Chromosome counts from populations throughout Alaska would allow future researchers to address whether hybridization between the diploids and polyploids is rare or common.

Within Alaska, we observed a few samples, just north of Anchorage, with genotypes that differed from remaining Alaskan samples. However, these patterns were not strong and they did not represent widespread clusters. Furthermore, we found no convincing genetic evidence for two clades (*B. neoalaskana* and *B. kenaica*) within Alaska. This is not to say that two such groups do not exist because such inferences are a function of sampling. We did not include samples from western and northern Alaska, and so it is possible that we have undersampled a clade.

### Historical Confusion and Implications for Wetland Ratings

Results of our study indicate a strong association between genetics and geography but poor correspondence between genetics and morphology. This has likely led to earlier taxonomic confusion, which has had implications for wetland ratings in Alaska. However, confusion has also been exacerbated by lags between taxonomic revisions, plant species databases, and systems for wetland ratings. Since 1988, when the initial list of wetland plants was developed for the purposes of determining hydrophytic vegetation (Reed, 1988), *Betula* in Alaska was treated as two species, *B. papyrifera* (with two varieties) and *B. kenaica*, following Hultén (1968). Wetland ratings on the NWPL are only assigned at the species level, so the two *B. papyrifera* varieties were treated as one species, and *B. kenaica* was treated separately with its own wetland rating. In 2012, the NWPL was using the Biota of North America Program (BONAP; Kartesz, 2015), a database that tracks plants occurrence records and nomenclature. At the time, BONAP included *Betula* only partially following the treatment of Furlow (1997). Since 2016, the NWPL has migrated from the BONAP to the PLANTS database (USDA NRCS, 2019). Thus, in 2012 *B*. *papyrifera* Marsh. subsp. *humilis* (Regel) Hultén was treated as *B. neoalaskana* Sargent and *B. papyrifera* var. *commutata* (Regel) Fernald as *B. papyrifera* Marsh, and both were treated separately at the species level for wetland ratings. *B. kenaica* Evans likewise was treated at the species level but was considered an upland plant on the NWPL until 2016. Hultén (1968) provided distribution maps for each of the taxa in his flora of Alaska. The database used by the NWPL in 2012, 2014, and 2016 only showed the taxa as state records or occurrences and did not show borough distributions. This gave the appearance that all three taxa were possibly spread throughout Alaska. This confusion exacerbated the problems of identifying birch trees in Alaska, and establishing wetland rating.

Our results clearly show that *B. papyrifera* is only found in southeast Alaska and east from there to eastern North America. However, we are unable to find consistent morphological features to distinguish this eastern group from the Western group found throughout most parts of Alaska. Furthermore, we detected no genetic distinctions between the two species found within Alaska: *B. kenaica* and *B. neoalaskana*. We suggest that morphological properties of these taxa are unreliable because their cryptic divergence may have been generated and maintained by cytogenetic differences.

## Conclusion

In conclusion, we find convincing genetic evidence, and indirect cytogenetic evidence that eastern *Betula (B. papyrifera)* is distinct from the tree species found throughout most of Alaska. We detected few putative hybrids between these groups, even in areas (of Alberta) where both groups were detected together. We have some evidence to suggest that *B. kenaica* and *B. neoalaskana* are similar genetically, but this will require additional studies to test. It is important to realize that plant distributions do not conform easily to geopolitical boundaries and this can have consequences when comparing Floras for different areas. In fact, it is very likely that *Betula* taxa in Alaska have much closer affinities with Eurasian populations, especially those in Siberia (Furlow, 1997). Future studies should therefore focus on possible relationships between birches within Alaska, adding samples from western and northern Alaska, and with populations from Eurasia, using molecular and morphological approaches, and chromosome counts.

## Data Availability Statement

The datasets generated for this study can be found in the NCBI Sequence Read Archive (accession PRJNA556994).

## Author Contributions

RL, PW, and CR conceived of the project and its design, and wrote and approved the manuscript. CR prepared genomic libraries and conducted all data analyses.

## Conflict of Interest

The authors declare that the research was conducted in the absence of any commercial or financial relationships that could be construed as a potential conflict of interest.
