# Supplementary material for: How Many Tree Species of Birch Are in Alaska? Implications for Wetland Designations
Source: Front Plant Sci. 2020 Jun 11;11:750. doi: 10.3389/fpls.2020.00750 (PMC7300271; doi:10.3389/fpls.2020.00750)
Supplement: Supplementary file 1 [file Table_1.DOCX]

Supplementary Table 1 – Sample locations

| Sample | Lead Collector | State or Province | lat deg | long deg |
| --- | --- | --- | --- | --- |
| 1028_1 | Wolf | AK | 64.01245 | -145.51803 |
| 1028_3 | Wolf | AK | 63.99394 | -145.51911 |
| 1028_4 | Wolf | AK | 63.98376 | -145.53539 |
| 1028_5 | Wolf | AK | 63.98411 | -145.54982 |
| 1029_1 | Wolf | AK | 61.34553 | -145.30808 |
| 1029_2 | Wolf | AK | 61.33172 | -145.31173 |
| 1029_3 | Wolf | AK | 61.32108 | -145.30834 |
| 1029_5 | Wolf | AK | 61.29307 | -145.27334 |
| 1030_1 | Wolf | AK | 61.79257 | -147.90414 |
| 1030_2 | Wolf | AK | 61.79816 | -147.98067 |
| 1030_4 | Wolf | AK | 61.80033 | -148.06033 |
| 1030_5 | Wolf | AK | 61.80445 | -148.08814 |
| 1031_1 | Wolf | AK | 60.48454 | -149.95335 |
| 1031_2 | Wolf | AK | 60.48823 | -149.97926 |
| 1031_3 | Wolf | AK | 60.48613 | -150.00458 |
| 1031_4 | Wolf | AK | 60.48814 | -150.02115 |
| 1031_5 | Wolf | AK | 60.48611 | -150.04754 |
| 8552a | Lichvar | AK | 61.27002214 | -149.8053185 |
| 8552b | Lichvar | AK | 61.27002214 | -149.8053185 |
| 8552c | Lichvar | AK | 61.27002214 | -149.8053185 |
| 8552d | Lichvar | AK | 61.27002214 | -149.8053185 |
| 8552e | Lichvar | AK | 61.27002214 | -149.8053185 |
| 8553a | Lichvar | AK | 61.27151854 | -149.7924457 |
| 8553b | Lichvar | AK | 61.27151854 | -149.7924457 |
| 8553c | Lichvar | AK | 61.27151854 | -149.7924457 |
| 8553d | Lichvar | AK | 61.27151854 | -149.7924457 |
| 8554a | Lichvar | AK | 61.29177842 | -149.7975521 |
| 8554b | Lichvar | AK | 61.29177842 | -149.7975521 |
| 8554c | Lichvar | AK | 61.29177842 | -149.7975521 |
| 8554d | Lichvar | AK | 61.29177842 | -149.7975521 |
| 8554e | Lichvar | AK | 61.29177842 | -149.7975521 |
| 8555a | Lichvar | AK | 61.18062486 | -149.8497813 |
| 8555b | Lichvar | AK | 61.18062486 | -149.8497813 |
| 8555c | Lichvar | AK | 61.18062486 | -149.8497813 |
| 8555d | Lichvar | AK | 61.18062486 | -149.8497813 |
| 8556a | Lichvar | AK | 61.235099 | -149.2723985 |
| 8556b | Lichvar | AK | 61.235099 | -149.2723985 |
| 8556c | Lichvar | AK | 61.235099 | -149.2723985 |
| 8556d | Lichvar | AK | 61.235099 | -149.2723985 |
| 8557a | Lichvar | AK | 61.32235388 | -149.5899057 |
| 8557d | Lichvar | AK | 61.32235388 | -149.5899057 |
| 8557e | Lichvar | AK | 61.32235388 | -149.5899057 |
| 8558a | Lichvar | AK | 61.37085105 | -149.508415 |
| 8558b | Lichvar | AK | 61.37085105 | -149.508415 |
| 8558c | Lichvar | AK | 61.37085105 | -149.508415 |
| 8558d | Lichvar | AK | 61.37085105 | -149.508415 |
| 8558e | Lichvar | AK | 61.37085105 | -149.508415 |
| 8559a | Lichvar | AK | 61.4032615 | -149.4570538 |
| 8559b | Lichvar | AK | 61.4032615 | -149.4570538 |
| 8559c | Lichvar | AK | 61.4032615 | -149.4570538 |
| 8559d | Lichvar | AK | 61.4032615 | -149.4570538 |
| 8559e | Lichvar | AK | 61.4032615 | -149.4570538 |
| 8560a | Lichvar | AK | 61.27645651 | -149.8251164 |
| 8560b | Lichvar | AK | 61.27645651 | -149.8251164 |
| 8560c | Lichvar | AK | 61.27645651 | -149.8251164 |
| 8560d | Lichvar | AK | 61.27645651 | -149.8251164 |
| 8560e | Lichvar | AK | 61.27645651 | -149.8251164 |
| 8561a | Lichvar | AK | 61.27645651 | -149.8251164 |
| 8561b | Lichvar | AK | 61.27645651 | -149.8251164 |
| 8561c | Lichvar | AK | 61.27645651 | -149.8251164 |
| 8561d | Lichvar | AK | 61.27645651 | -149.8251164 |
| 8561e | Lichvar | AK | 61.27645651 | -149.8251164 |
| 8562b | Lichvar | AK | 61.46231521 | -149.3564911 |
| 8562c | Lichvar | AK | 61.46231521 | -149.3564911 |
| 8562d | Lichvar | AK | 61.46231521 | -149.3564911 |
| 8562e | Lichvar | AK | 61.46231521 | -149.3564911 |
| 8563a | Lichvar | AK | 61.77470164 | -148.4942851 |
| 8563b | Lichvar | AK | 61.77470164 | -148.4942851 |
| 8563c | Lichvar | AK | 61.77470164 | -148.4942851 |
| 8563d | Lichvar | AK | 61.77470164 | -148.4942851 |
| 8563e | Lichvar | AK | 61.77470164 | -148.4942851 |
| 8564a | Lichvar | AK | 61.78999999 | -148.4500042 |
| 8564b | Lichvar | AK | 61.78999999 | -148.4500042 |
| 8564c | Lichvar | AK | 61.78999999 | -148.4500042 |
| 8564d | Lichvar | AK | 61.78999999 | -148.4500042 |
| 8565a | Lichvar | AK | 62.29662289 | -150.0778865 |
| 8565b | Lichvar | AK | 62.29662289 | -150.0778865 |
| 8565c | Lichvar | AK | 62.29662289 | -150.0778865 |
| 8565d | Lichvar | AK | 62.29662289 | -150.0778865 |
| 8565e | Lichvar | AK | 62.29662289 | -150.0778865 |
| 8566a | Lichvar | AK | 62.59198207 | -150.239728 |
| 8566d | Lichvar | AK | 62.59198207 | -150.239728 |
| 8566e | Lichvar | AK | 62.59198207 | -150.239728 |
| 8567a | Lichvar | AK | 62.87575933 | -155.8223199 |
| 8567b | Lichvar | AK | 62.87575933 | -155.8223199 |
| 8567c | Lichvar | AK | 62.87575933 | -155.8223199 |
| 8568b | Lichvar | AK | 64.7025893 | -148.6575603 |
| 8568c | Lichvar | AK | 64.7025893 | -148.6575603 |
| 8568d | Lichvar | AK | 64.7025893 | -148.6575603 |
| 8568e | Lichvar | AK | 64.7025893 | -148.6575603 |
| 8569a | Lichvar | AK | 64.73114621 | -147.3266014 |
| 8569b | Lichvar | AK | 64.73114621 | -147.3266014 |
| 8569c | Lichvar | AK | 64.73114621 | -147.3266014 |
| 8569d | Lichvar | AK | 64.73114621 | -147.3266014 |
| 8569e | Lichvar | AK | 64.73114621 | -147.3266014 |
| 8570a | Lichvar | AK | 64.53124094 | -147.0080239 |
| 8570b | Lichvar | AK | 64.53124094 | -147.0080239 |
| 8570c | Lichvar | AK | 64.53124094 | -147.0080239 |
| 8570d | Lichvar | AK | 64.53124094 | -147.0080239 |
| 8570e | Lichvar | AK | 64.53124094 | -147.0080239 |
| 8571a | Lichvar | AK | 64.41769003 | -146.8954768 |
| 8571b | Lichvar | AK | 64.41769003 | -146.8954768 |
| 8571c | Lichvar | AK | 64.41769003 | -146.8954768 |
| 8571d | Lichvar | AK | 64.41769003 | -146.8954768 |
| 8571e | Lichvar | AK | 64.41769003 | -146.8954768 |
| 8572a | Lichvar | AK | 64.91364107 | -147.7051015 |
| 8572b | Lichvar | AK | 64.91364107 | -147.7051015 |
| 8572c | Lichvar | AK | 64.91364107 | -147.7051015 |
| 8572d | Lichvar | AK | 64.91364107 | -147.7051015 |
| 8572e | Lichvar | AK | 64.91364107 | -147.7051015 |
| 8573a | Lichvar | AK | 65.03701084 | -147.4564558 |
| 8573b | Lichvar | AK | 65.03701084 | -147.4564558 |
| 8573c | Lichvar | AK | 65.03701084 | -147.4564558 |
| 8573e | Lichvar | AK | 65.03701084 | -147.4564558 |
| 8574a | Lichvar | AK | 65.15581255 | -147.3451886 |
| 8574b | Lichvar | AK | 65.15581255 | -147.3451886 |
| 8574c | Lichvar | AK | 65.15581255 | -147.3451886 |
| 8574d | Lichvar | AK | 65.15581255 | -147.3451886 |
| 8574e | Lichvar | AK | 65.15581255 | -147.3451886 |
| 8575a | Lichvar | AK | 65.2600535 | -146.7706736 |
| 8575b | Lichvar | AK | 65.2600535 | -146.7706736 |
| 8575c | Lichvar | AK | 65.2600535 | -146.7706736 |
| 8575d | Lichvar | AK | 65.2600535 | -146.7706736 |
| 8575e | Lichvar | AK | 65.2600535 | -146.7706736 |
| 8576a | Lichvar | AK | 60.44246476 | -149.979909 |
| 8576b | Lichvar | AK | 60.44246476 | -149.979909 |
| 8576c | Lichvar | AK | 60.44246476 | -149.979909 |
| 8576d | Lichvar | AK | 60.44246476 | -149.979909 |
| 8576e | Lichvar | AK | 60.44246476 | -149.979909 |
| 8577a | Lichvar | AK | 60.4553066 | -149.9745739 |
| 8577b | Lichvar | AK | 60.4553066 | -149.9745739 |
| 8577c | Lichvar | AK | 60.4553066 | -149.9745739 |
| 8577d | Lichvar | AK | 60.4553066 | -149.9745739 |
| 8577e | Lichvar | AK | 60.4553066 | -149.9745739 |
| 8578a | Lichvar | AK | 60.48392541 | -149.9522521 |
| 8578b | Lichvar | AK | 60.48392541 | -149.9522521 |
| 8578c | Lichvar | AK | 60.48392541 | -149.9522521 |
| 8578d | Lichvar | AK | 60.48392541 | -149.9522521 |
| 8578e | Lichvar | AK | 60.48392541 | -149.9522521 |
| ALB_1 | Rai | Alberta | 53.799167 | -114.043889 |
| ALB_2 | Rai | Alberta | 53.7975 | -114.062778 |
| ALB_3 | Rai | Alberta | 53.774722 | -114.043889 |
| ALB_4 | Rai | Alberta | 53.781111 | -114.048333 |
| ALB_5 | Rai | Alberta | 53.832222 | -114.068333 |
| ALB_6 | Rai | Alberta | 53.856389 | -114.051389 |
| ALB_7 | Rai | Alberta | 53.862222 | -114.051389 |
| ALB_8 | Rai | Alberta | 53.878889 | -114.051389 |
| BP_02 | Surdyk | AK | 59.45275333 | -135.3134781 |
| BP_03 | Surdyk | AK | 59.45275333 | -135.3134781 |
| BP_04 | Surdyk | AK | 59.45275333 | -135.3134781 |
| BP_05 | Surdyk | AK | 59.45275333 | -135.3134781 |
| BP_06 | Surdyk | AK | 59.45634958 | -135.3264238 |
| BP_07 | Surdyk | AK | 59.45634958 | -135.3264238 |
| BP_08 | Surdyk | AK | 59.45634958 | -135.3264238 |
| BP_09 | Surdyk | AK | 59.45634958 | -135.3264238 |
| BP_10 | Surdyk | AK | 59.45634958 | -135.3264238 |
| BP_13 | Surdyk | AK | 59.47451509 | -135.2887588 |
| BP_14 | Surdyk | AK | 59.47451509 | -135.2887588 |
| BP_16 | Belt | AK | 59.49877221 | -135.3603027 |
| BP_17 | Belt | AK | 59.49795412 | -135.3606296 |
| BP_18 | Belt | AK | 59.49779565 | -135.3594799 |
| BP_19 | Belt | AK | 59.49759819 | -135.3594425 |
| BP_20 | Belt | AK | 59.48273269 | -135.3474566 |
| BP_21 | Belt | AK | 59.48421754 | -135.3462713 |
| BP_22 | Belt | AK | 59.48526074 | -135.3456994 |
| BP_24 | Belt | AK | 59.49752431 | -135.3601835 |
| BP_25 | Belt | AK | 59.48384871 | -135.3465147 |
| Ken1 | Barnes | AK | 61.2853544 | -142.525999 |
| Ken2 | Barnes | AK | 61.2853 | -142.5302 |
| MNSE_01 | Eggers | MN | 46.179433 | -94.104531 |
| MNSE_02 | Eggers | MN | 46.5366 | -93.401202 |
| MNSE_03 | Eggers | MN | 46.646272 | -93.465049 |
| MNSE_04 | Eggers | MN | 46.539257 | -93.338116 |
| MNSE_05 | Eggers | MN | 46.277439 | -94.142414 |
| MNSE_06 | Eggers | MN | 46.116325 | -93.732366 |
| MNSE_07 | Eggers | MN | 46.330359 | -94.130806 |
| NB_1 | Goulet | NH | 43.75077 | -72.36027 |
| NB_2 | Goulet | NH | 43.79539 | -72.39986 |
| NB_3 | Goulet | NH | 43.78366 | -72.39396 |
| NB_4 | Goulet | NH | 43.7656 | -72.37616 |
| NB_5 | Goulet | NH | 43.74395 | -72.34621 |
