# Supplementary material for: How Many Tree Species of Birch Are in Alaska? Implications for Wetland Designations
Source: Front Plant Sci. 2020 Jun 11;11:750. doi: 10.3389/fpls.2020.00750 (PMC7300271; doi:10.3389/fpls.2020.00750)
Supplement: Supplementary file 2 [file Table_2.DOCX]

Supplementary table 2. Structure grouping and inferred ploidy for each sample of Betula

| Population | Sample | Group | Het loci | Ploidy |
| --- | --- | --- | --- | --- |
| 8554 | 8554a | Western | 587 | DIPLOID |
| 8554 | 8554b | Western | 602 | DIPLOID |
| 8554 | 8554c | Western | 431 | DIPLOID |
| 8554 | 8554d | Western | 585 | DIPLOID |
| 8554 | 8554e | Western | 606 | DIPLOID |
| 8552 | 8552a | Western | 481 | DIPLOID |
| 8552 | 8552b | Western | 624 | DIPLOID |
| 8552 | 8552c | Western | 597 | DIPLOID |
| 8552 | 8552d | Western | 596 | DIPLOID |
| 8552 | 8552e | Western | 549 | DIPLOID |
| 1028 | 1028_1 | Western | 605 | DIPLOID |
| 1028 | 1028_3 | Western | 588 | DIPLOID |
| 1028 | 1028_4 | Western | 375 | DIPLOID |
| 1028 | 1028_5 | Western | 508 | DIPLOID |
| 1029 | 1029_1 | Western | 659 | DIPLOID |
| 1029 | 1029_2 | Western | 200 | DIPLOID |
| 1029 | 1029_3 | Western | 687 | DIPLOID |
| 1029 | 1029_5 | Western | 557 | DIPLOID |
| 1030 | 1030_1 | Western | 644 | DIPLOID |
| 1030 | 1030_2 | Western | 630 | DIPLOID |
| 1030 | 1030_4 | Western | 379 | DIPLOID |
| 1030 | 1030_5 | Western | 665 | DIPLOID |
| 1031 | 1031_1 | Western | 611 | DIPLOID |
| 1031 | 1031_2 | Western | 475 | DIPLOID |
| 1031 | 1031_3 | Western | 638 | DIPLOID |
| 1031 | 1031_4 | Western | 616 | DIPLOID |
| 1031 | 1031_5 | Western | 619 | DIPLOID |
| 8553 | 8553a | Western | 608 | DIPLOID |
| 8553 | 8553c | Western | 495 | AMBIGUOUS |
| 8553 | 8553d | Western | 572 | DIPLOID |
| 8555 | 8555a | Western | 198 | DIPLOID |
| 8555 | 8555b | Western | 656 | DIPLOID |
| 8555 | 8555c | Western | 620 | DIPLOID |
| 8555 | 8555d | Western | 572 | AMBIGUOUS |
| 8556 | 8556a | Western | 295 | DIPLOID |
| 8556 | 8556b | Western | 618 | DIPLOID |
| 8556 | 8556c | Western | 529 | DIPLOID |
| 8556 | 8556d | Western | 600 | DIPLOID |
| 8557 | 8557a | Western | 612 | DIPLOID |
| 8557 | 8557d | Western | 334 | DIPLOID |
| 8557 | 8557e | Western | 558 | DIPLOID |
| 8558 | 8558a | Western | 642 | DIPLOID |
| 8558 | 8558b | Western | 627 | DIPLOID |
| 8558 | 8558c | Western | 317 | DIPLOID |
| 8558 | 8558d | Western | 582 | DIPLOID |
| 8558 | 8558e | Western | 564 | DIPLOID |
| 8559 | 8559a | Western | 569 | DIPLOID |
| 8559 | 8559b | Western | 566 | DIPLOID |
| 8559 | 8559c | Western | 547 | DIPLOID |
| 8559 | 8559d | Western | 623 | DIPLOID |
| 8559 | 8559e | Western | 572 | DIPLOID |
| 8560 | 8560a | Western | 552 | DIPLOID |
| 8560 | 8560b | Western | 573 | DIPLOID |
| 8560 | 8560c | Western | 655 | DIPLOID |
| 8560 | 8560d | Western | 563 | DIPLOID |
| 8560 | 8560e | Western | 610 | DIPLOID |
| 8561 | 8561a | Western | 349 | DIPLOID |
| 8561 | 8561b | Western | 591 | DIPLOID |
| 8561 | 8561c | Western | 613 | DIPLOID |
| 8561 | 8561d | Western | 491 | DIPLOID |
| 8561 | 8561e | Western | 615 | DIPLOID |
| 8562 | 8562b | Western | 560 | DIPLOID |
| 8562 | 8562c | Western | 65 | AMBIGUOUS |
| 8562 | 8562d | Western | 526 | DIPLOID |
| 8562 | 8562e | Western | 632 | DIPLOID |
| 8563 | 8563a | Western | 340 | DIPLOID |
| 8563 | 8563b | Western | 542 | DIPLOID |
| 8563 | 8563c | Western | 444 | DIPLOID |
| 8563 | 8563d | Western | 533 | DIPLOID |
| 8563 | 8563e | Western | 605 | DIPLOID |
| 8564 | 8564a | Western | 517 | DIPLOID |
| 8564 | 8564b | Western | 112 | DIPLOID |
| 8564 | 8564c | Western | 482 | DIPLOID |
| 8564 | 8564d | Western | 627 | DIPLOID |
| 8565 | 8565a | Western | 560 | DIPLOID |
| 8565 | 8565b | Western | 572 | DIPLOID |
| 8565 | 8565c | Western | 568 | DIPLOID |
| 8565 | 8565d | Western | 581 | DIPLOID |
| 8565 | 8565e | Western | 296 | DIPLOID |
| 8566 | 8566a | Western | 462 | DIPLOID |
| 8566 | 8566d | Western | 359 | DIPLOID |
| 8566 | 8566e | Western | 637 | DIPLOID |
| 8567 | 8567a | Western | 551 | DIPLOID |
| 8567 | 8567b | Western | 600 | DIPLOID |
| 8567 | 8567c | Western | 616 | DIPLOID |
| 8568 | 8568b | Western | 467 | DIPLOID |
| 8568 | 8568c | Western | 515 | DIPLOID |
| 8568 | 8568d | Western | 591 | DIPLOID |
| 8568 | 8568e | Western | 571 | DIPLOID |
| 8569 | 8569a | Western | 474 | DIPLOID |
| 8569 | 8569b | Western | 650 | AMBIGUOUS |
| 8569 | 8569c | Western | 580 | DIPLOID |
| 8569 | 8569d | Western | 568 | DIPLOID |
| 8569 | 8569e | Western | 560 | DIPLOID |
| 8570 | 8570a | Western | 643 | DIPLOID |
| 8570 | 8570b | Western | 568 | DIPLOID |
| 8570 | 8570c | Western | 518 | DIPLOID |
| 8570 | 8570d | Western | 550 | DIPLOID |
| 8570 | 8570e | Western | 596 | DIPLOID |
| 8571 | 8571a | Western | 197 | DIPLOID |
| 8571 | 8571b | Western | 628 | DIPLOID |
| 8571 | 8571c | Western | 547 | DIPLOID |
| 8571 | 8571d | Western | 584 | DIPLOID |
| 8571 | 8571e | Western | 602 | DIPLOID |
| 8572 | 8572a | Western | 474 | DIPLOID |
| 8572 | 8572b | Western | 612 | DIPLOID |
| 8572 | 8572c | Western | 400 | DIPLOID |
| 8572 | 8572d | Western | 676 | DIPLOID |
| 8572 | 8572e | Western | 577 | DIPLOID |
| 8573 | 8573a | Western | 606 | DIPLOID |
| 8573 | 8573b | Western | 624 | DIPLOID |
| 8573 | 8573c | Western | 66 | DIPLOID |
| 8573 | 8573e | Western | 612 | DIPLOID |
| 8574 | 8574a | Western | 472 | DIPLOID |
| 8574 | 8574b | Western | 560 | DIPLOID |
| 8574 | 8574c | Western | 629 | DIPLOID |
| 8574 | 8574d | Western | 604 | DIPLOID |
| 8574 | 8574e | Western | 549 | DIPLOID |
| 8575 | 8575a | Western | 582 | DIPLOID |
| 8575 | 8575b | Western | 652 | DIPLOID |
| 8575 | 8575c | Western | 614 | DIPLOID |
| 8575 | 8575d | Western | 544 | DIPLOID |
| 8575 | 8575e | Western | 596 | DIPLOID |
| 8576 | 8576a | Western | 473 | DIPLOID |
| 8576 | 8576b | Western | 555 | DIPLOID |
| 8576 | 8576c | Western | 495 | DIPLOID |
| 8576 | 8576d | Western | 596 | DIPLOID |
| 8576 | 8576e | Western | 572 | DIPLOID |
| 8577 | 8577a | Western | 403 | DIPLOID |
| 8577 | 8577b | Western | 633 | DIPLOID |
| 8577 | 8577c | Western | 424 | DIPLOID |
| 8577 | 8577d | Western | 554 | DIPLOID |
| 8577 | 8577e | Western | 491 | DIPLOID |
| 8578 | 8578a | Western | 625 | DIPLOID |
| 8578 | 8578b | Western | 638 | DIPLOID |
| 8578 | 8578c | Western | 354 | DIPLOID |
| 8578 | 8578d | Western | 541 | DIPLOID |
| 8578 | 8578e | Western | 552 | DIPLOID |
| Ken | Ken1 | Western | 638 | DIPLOID |
| Ken | Ken2 | Western | 646 | DIPLOID |
| 8553 | 8553b | Eastern | 269 | AMBIGUOUS |
| ALB | ALB_4 | Western | 579 | DIPLOID |
| ALB | ALB_6 | Western | 617 | DIPLOID |
| ALB | ALB_7 | Western | 597 | DIPLOID |
| ALB | ALB_8 | Western | 108 | DIPLOID |
| ALB | ALB_1 | Eastern | 828 | AMBIGUOUS |
| ALB | ALB_2 | Eastern | 823 | NOT_DIPLOID |
| ALB | ALB_3 | Eastern | 848 | NOT_DIPLOID |
| ALB | ALB_5 | Eastern | 611 | AMBIGUOUS |
| MNSE | MNSE_01 | Eastern | 897 | NOT_DIPLOID |
| MNSE | MNSE_02 | Eastern | 931 | NOT_DIPLOID |
| MNSE | MNSE_03 | Eastern | 717 | AMBIGUOUS |
| MNSE | MNSE_04 | Eastern | 818 | NOT_DIPLOID |
| MNSE | MNSE_05 | Eastern | 578 | AMBIGUOUS |
| MNSE | MNSE_06 | Eastern | 884 | NOT_DIPLOID |
| MNSE | MNSE_07 | Eastern | 874 | NOT_DIPLOID |
| NB | NB_1 | Eastern | 761 | AMBIGUOUS |
| NB | NB_2 | Eastern | 553 | NOT_DIPLOID |
| NB | NB_3 | Eastern | 806 | AMBIGUOUS |
| NB | NB_4 | Eastern | 879 | NOT_DIPLOID |
| NB | NB_5 | Eastern | 785 | NOT_DIPLOID |
| BP | BP_02 | Eastern | 865 | NOT_DIPLOID |
| BP | BP_03 | Eastern | 234 | NOT_DIPLOID |
| BP | BP_04 | Eastern | 407 | NOT_DIPLOID |
| BP | BP_05 | Eastern | 740 | NOT_DIPLOID |
| BP | BP_06 | Eastern | 578 | AMBIGUOUS |
| BP | BP_07 | Eastern | 479 | NOT_DIPLOID |
| BP | BP_08 | Eastern | 722 | NOT_DIPLOID |
| BP | BP_09 | Eastern | 798 | NOT_DIPLOID |
| BP | BP_10 | Eastern | 888 | NOT_DIPLOID |
| BP | BP_13 | Eastern | 791 | NOT_DIPLOID |
| BP | BP_14 | Eastern | 742 | AMBIGUOUS |
| BP | BP_16 | Eastern | 794 | AMBIGUOUS |
| BP | BP_17 | Eastern | 911 | NOT_DIPLOID |
| BP | BP_18 | Eastern | 776 | NOT_DIPLOID |
| BP | BP_19 | Eastern | 817 | NOT_DIPLOID |
| BP | BP_20 | Eastern | 832 | NOT_DIPLOID |
| BP | BP_21 | Eastern | 706 | NOT_DIPLOID |
| BP | BP_22 | Eastern | 477 | NOT_DIPLOID |
| BP | BP_24 | Eastern | 879 | NOT_DIPLOID |
| BP | BP_25 | Eastern | 511 | AMBIGUOUS |
